# PCL-Coated Multi-Substituted Calcium Phosphate Bone Scaffolds with Enhanced Properties

**DOI:** 10.3390/ma14164403

**Published:** 2021-08-06

**Authors:** Leonard Bauer, Maja Antunović, Gloria Gallego-Ferrer, Marica Ivanković, Hrvoje Ivanković

**Affiliations:** 1 Faculty of Chemical Engineering and Technology, University of Zagreb, HR-10001 Zagreb, Croatia; maja.antunovic2007@gmail.com (M.A.); mivank@fkit.hr (M.I.); hivan@fkit.hr (H.I.); 2 Centre for Biomaterials and Tissue Engineering (CBIT), Universitat Politècnica de València, 46022 Valencia, Spain; ggallego@ter.upv.es; 3 Biomedical Research Networking Center in Bioengineering, Biomaterials and Nanomedicine (CIBER-BBN), 46022 Valencia, Spain

**Keywords:** scaffold, hydroxyapatite, whitlockite, Mg, Sr, co-substitution, poly(*ε*-caprolactone), hMSCs, osteogenic differentiation

## Abstract

Ionic substitutions within the hydroxyapatite lattice are a widely used approach to mimic the chemical composition of the bone mineral. In this work, Sr-substituted and Mg- and Sr-co-substituted calcium phosphate (CaP) scaffolds, with various levels of strontium and magnesium substitution, were prepared using the hydrothermal method at 200 °C. Calcium carbonate skeletons of cuttlefish bone, ammonium dihydrogenphosphate (NH_4_H_2_PO_4_), strontium nitrate (Sr(NO_3_)_2_), and magnesium perchlorate (Mg(ClO_4_)_2_) were used as reagents. Materials were characterized by means of X-ray diffraction (XRD), Fourier transform infrared spectroscopy (FTIR) and scanning electron microscopy (SEM). Whole powder pattern decomposition refinements of XRD data indicated that increased magnesium content in the Mg- and Sr-co-substituted scaffolds was related to an increased proportion of the whitlockite (WH) phase in the biphasic hydroxyapatite (HAp)/WH scaffolds. In addition, refinements indicate that Sr^2+^ ions have replaced Ca^2+^ sites in the WH phase. Furthermore, PCL-coated Mg-substituted and Sr- and Mg-co-substituted scaffolds, with the HAp:WH wt. ratio of 90:10 were prepared by vacuum impregnation. Results of compression tests showed a positive impact of the WH phase and PCL coating on the mechanical properties of scaffolds. Human mesenchymal stem cells (hMSCs) were cultured on composite scaffolds in an osteogenic medium for 21 days. Immunohistochemical staining showed that Mg-Sr-CaP/PCL scaffold exhibited higher expression of collagen type I than the Mg-CaP/PCL scaffold, indicating the positive effect of Sr^2+^ ions on the differentiation of hMSCs, in concordance with histology results. Reverse transcription-quantitative polymerase chain reaction (RT-qPCR) analysis confirmed an early stage of osteogenic differentiation.

## 1. Introduction

Synthetic calcium phosphates (CaPs), in particular hydroxyapatite (HAp, Ca_10_(PO_4_)_6_(OH)_2_, Ca/P = 1.667), are the most commonly used ceramics in dentistry, orthopedics and bone tissue engineering. The major challenge in bone tissue engineering is to design a porous scaffold that mimics the three-dimensional architecture and intrinsic properties of natural bone.

Natural bone mineral differs from stoichiometric hydroxyapatite owing to the anionic and/or cationic substitutions within the apatite lattice. For this reason, today, ionic substitutions within the apatite lattice are one of the most widely used approaches to mimic the chemical composition of the bone mineral and to improve the biological performance of calcium phosphate materials. Several reviews have been published on the preparation of ion substituted calcium phosphate materials [1,2,3,4]. There are many studies on the individual substitution of several ions such as Mg^2+^ [5,6,7,8] and Sr^2+^ [9,10,11,12], into HAp, meanwhile, simultaneous incorporation of two or more elements into HAp is less studied.

The interest for the strontium substitution into calcium phosphates increased after strontium ranelate was found to enhance osteoblast differentiation, stimulating bone formation, and slow down the resorption activity and differentiation of osteoclasts, thus, reducing the bone degradation rate [13,14]. Sr^2+^ ion within the hydroxyapatite structure substitutes Ca^2+^ in the entire substitution range to a strontium apatite while unit cell parameters and volume increase almost linearly [15,16].

Magnesium is one of the main substituents in biological apatites (0.44 wt.% in enamel, 1.23 wt.% in dentin, 0.72 wt.% in bone) [17]. It is also required for osteoporosis prevention and bone tissue damage, stimulates growth and development of bones and influences vitamin D secretion. The incorporation of magnesium into synthetic HAp is limited (up to 10 wt.%), the main portion being surface-bound or present in a separate phase [18,19].

The co-substitution of two different ions, with balancing differences in ionic radius and/or valence can promote ion incorporation. Strontium, an ion of a larger radius than calcium, can support the substitution of magnesium ion that has a smaller ionic radius than calcium. There are no many studies that consider the simultaneous incorporation of Sr^2+^ and Mg^2+^ ions into the structure of hydroxyapatite.

Aina et al. [20] synthesized magnesium- and strontium-co-substituted hydroxyapatite by an aqueous precipitation method. They obtained biphasic calcium phosphates (BCPs) with variable HAp/*β*-tricalcium phosphate (*β*-Ca_3_(PO_4_)_2_, *β*-TCP) ratios. Geng et al. [21] synthesized a series of Sr/Mg-co-substituted HAp-s by a hydrothermal method. The in vitro results showed that the co-addition can result in both, increased and decreased cell proliferation and bioactivity, as compared to HAp, depending on the ion concentration [21].

Considering the importance of Sr^2+^ and Mg^2+^ in the bone growth process, a further focus on the effect of Sr and Mg co-substitution on the structural, mechanical and biological properties of calcium phosphate ceramics are of great interest.

This study is a continuation of our previous work [22] where bone mimetic highly porous Mg-substituted calcium phosphate scaffolds were synthesized by hydrothermal method, using calcium carbonate skeletons of cuttlefish bone. In this work, Sr-substituted and Mg- and Sr-co-substituted calcium phosphate scaffolds, with various levels of strontium and magnesium substitution were prepared using the hydrothermal method. Furthermore, to improve mechanical properties of the scaffolds, characterized by the brittleness and low fracture strength in load-bearing applications, new composite scaffolds, combining biodegradable polymer-polycaprolactone (PCL) with ion-substituted scaffolds were fabricated. The effect of ion substitution and the polymer coating on the mechanical and biological properties of the composite scaffolds has been investigated.

## 2. Materials and Methods

### 2.1. Scaffold Preparation

Cuttlefish bones (*Sepia officinalis* L.) from the Adriatic Sea were used as a calcium source for the hydrothermal synthesis of ion-substituted calcium phosphates. The bone was treated as previously described [22]. In short, a dorsal shield was mechanically removed from the cuttlefish bones. Lamellae matrix of the bones was cut into pieces (≈2 cm^3^) and treated with an aqueous solution of sodium hypochlorite (NaClO, 13% active chlorine, Gram-mol, Zagreb, Croatia) at room temperature for 48 h and repeatedly washed with boiling demineralized water to remove the organic component. Pieces of cuttlefish bone were sealed with the required quantity of a 0.6 mol dm^−3^ aqueous solution of ammonium dihydrogenphosphate (NH_4_H_2_PO_4_, 99% Fisher Scientific, Leicester, UK), magnesium perchlorate (Mg(ClO_4_)_2_, 99% Sigma Aldrich, Steinheim, Germany) and/or strontium nitrate, (Sr(NO_3_)_2_, 99%, ACS reagent, Acros Organics, Geel, Belgium) in TEFLON lined stainless steel pressure vessels at 200 °C for 48 h. The self-generated pressure inside the reactor reached up to 20 bars. After hydrothermal treatment, the resulting pieces of scaffolds were washed with boiling demineralized water, dried at 105 °C and stored for further analysis. All calculations were based on the nominal composition of substituted hydroxyapatite, with the constant (Ca + Mg + Sr)/P molar ratio of 1.667. Four samples with different Sr/(Ca + Sr) molar ratio (0.01, 0.025, 0.05 and 0.10) were prepared and labelled as 1-Sr-CaP, 2.5-Sr-CaP, 5-Sr-CaP and 10-Sr-CaP, respectively. The number before Sr-CaP is the nominal Sr mol.% value expected to be substituted into hydroxyapatite (in place of Ca) (Table 1). Furthermore, four co-substituted samples with different Mg/(Ca + Mg + Sr) and Sr/(Ca + Mg + Sr) molar ratios (see Table 1) were prepared as well.

### 2.2. XRD Analysis and Rietveld Refinement

Samples were characterized with the X-ray powder diffraction (XRD Shimadzu 6000, Shimadzu Corporation, Tokyo, Japan) with Bragg–Brentano geometry and CuKα source. Samples were scanned in the range of diffraction angles between 5 and 70 with a 2*θ* step of 0.02 × 2 s^−1^ for qualitative analysis, and 0.02 × 10 s^−1^ for HAp structure refinement (with the known addition of silicon standard, NIST SRM 640e, Gaithersburg, MD, USA). Crystal phase identification was performed by comparing the experimental XRD patterns to standards compiled by the International Centre for Diffraction Data (ICDD, Newtown Square, PA, USA) using the reference cards for HAp, whitlockite and aragonite (09-432, 70-2064 and 41-1475, respectively).

The whole powder pattern decomposition refinements and the Rietveld structure refinement were carried out using a program DIFFRAC.SUITE TOPAS v.5.0 (Bruker, Karlsruhe, Germany) [23]. The Chebyshev polynomial equation of the 5th order was used to describe the background. The peak profiles were fitted with pseudo-Voigt function. The structure of Holly Springs HAp, without the inclusion of CO_3_^2−^ in the structure [24], was used as a starting model for the Rietveld structure refinement. Unit cell parameters, positional parameters and occupancies were varied [25]. The structure of synthetic whitlockite, Ca_18_Mg_2_(HPO_4_)_2_(PO_4_)_12_, refined by Gopal [26] and structure of aragonite [27] were used as models for remaining crystal phases.

### 2.3. FTIR Analysis

Fourier transform infrared spectra (FTIR) was performed by attenuated total reflectance (ATR) spectroscopy of solids with a diamond crystal (Bruker Vertex 70, Bruker Optik GmbH, Ettlingen, Germany) at 20 °C over the spectral range of 400 to 4000 cm^−1^, with 32 scans and 4 cm^−1^ resolution.

### 2.4. SEM-EDX Analysis

The morphology of scaffolds was examined by scanning electron microscopy (SEM Tescan Vega III Easyprobe, Tescan Orsay Holding, Brno, Czech Republic). An energy-dispersive X-ray (EDX) spectrometer connected to the SEM was used to determine the elemental composition of scaffolds. Prior to the SEM analysis, the samples were sputtered with gold and palladium for 120 s.

### 2.5. MTT Viability Assay

3-(4,5-dimethylthiazol-2-yl)-2,5-diphenyltetrazolium bromide (MTT, Sigma-Aldrich, Steinheim, Germany) assay was used to determine the potential cytotoxic effect of prepared scaffolds. The assay was performed using a human embryonic kidney (Hek293) cell line, kindly provided by Prof. Inga Urlić, Faculty of Science, University of Zagreb, by indirect contact cell culture for 72 h as previously described [22]. A fresh growth medium was added to untreated control cells as well, which were defined as 100% viable. The percentage of viable cells cultured with sample extracts was calculated with respect to untreated cells.

Briefly, powder of samples were disinfected with alcohol (96% *w*/*v* ethanol, Kefo, Sisak, Croatia), extensively washed with sterile phosphate-buffered saline (PBS, Sigma-Aldrich, Steinheim, Germany). Disinfected samples were resuspended in Dulbecco’s modified Eagle culture medium with 4500 mg L^−1^ glucose (DMEM-high glucose, Lonza, Basel, Switzerland) at a concentration of 10 mg mL^−1^ and incubated at 4 °C for 7 days. After the incubation period, centrifuged extracts were used as media for viability testing.

### 2.6. Preparation of Composite Scaffolds

Unsubstituted (CaP) scaffold, Mg-substituted scaffold 1-Mg-CaP, prepared in previous work [22], and Sr and Mg-co-substituted scaffolds, 1-Mg-1-Sr-CaP and 1-Mg-5-Sr-CaP were impregnated with PCL solution in chloroform using the vacuum impregnation unit (CitoVac, Struers, Cleveland, OH, USA). Vacuum, which removes the air and ensures that polymer enters into cuttlefish bone-derived scaffolds, was used for the impregnation process by our research group on similar samples [28,29].

A homogeneous 10% *w*/*v* solution of poly(*ε*-caprolactone) (PCL, Mn = 45,000, Sigma-Aldrich, Steinheim, Germany) was prepared by an intensive stirring of PCL pellets in chloroform (CHCl_3_, p.a., Kemika, Zagreb, Croatia). PCL solution is poured into the graduated cylinder and connected to the vacuum chamber through the tube. The specimens of porous CaP scaffolds were put in a beaker and placed in the vacuum chamber. The vacuum was set to 0.11 bars. After 10 min at achieved pressure, the valve was open to suck the PCL solution through the tube filling the beaker over the porous specimens. The specimens were soaked in the polymer solution for 10 min. Afterward, the vacuum was stopped to allow the air pressure to force the PCL solution into the pores of the specimens. The beaker was removed from the vacuum chamber. The soaked scaffolds were put on a net and placed in the vacuum chamber again. The vacuum was restored to remove the excess PCL solution away from the scaffolds and to dry the specimens.

### 2.7. Mechanical Testing

Mechanical properties of selected scaffolds were determined by a static compression test. The Microtest standard instrument (Microtest S.A., Madrid, Spain) with a 500 N loading cell and a crosshead velocity of 0.2 mm min^−1^ was used to examine samples by dry test. Scaffolds dimensions were at around 5 × 5 × 5 mm and at least nine scaffold replicas were used to overcome the uncertainty and randomness of samples derived from biogenic sources. With the same intention, the compressive load was applied perpendicular to the lamellae of each specimen. The elastic compression modulus was determined as the slope of the initial linear range of stress-strain curves.

### 2.8. Osteogenic Differentiation of hMSCs Cultured in 3D Static Conditions

hMSCs cells were kindly provided by Prof. Inga Urlić, Faculty of Science, University of Zagreb. Primary isolation of hMSCs from bone marrow aspirates and cell culture of hMSCs on prepared scaffolds under static conditions were performed as described in detail earlier [30,31].

Prior to the cell seeding, scaffolds were disinfected with ethanol (96% *w*/*v*, Kefo, Sisak, Croatia) for 4 h and sterilized under UV light. The cells were seeded on the scaffolds (5 mm in diameter) at a density of 5 × 10^5^ cells 200 µL^−1^ of proliferation medium in tissue-culture non-treated 96-well plates (Sarsted, Nümbrecht, Germany) in triplicates. Following 24 h, the proliferation medium was changed to the osteogenic induction medium containing Minimum Essential Medium-Alpha Eagle (α-MEM, Lonza, Basel, Switzerland), 10% FBS, 1% penicillin/streptomycin, 50 µg mL^−1^ ascorbic acid (Sigma-Aldrich, Steinheim, Germany), 4 mmol L^−1^ *β*-glycerophosphate (Sigma-Aldrich, Steinheim, Germany) and 1 × 10^7^ mol L^−1^ dexamethasone (Sigma-Aldrich, Steinheim, Germany). The medium was exchanged every 2 days for 21 days.

### 2.9. Histological Analysis

The tissues were processed in a routine manner as described earlier [32]. Tissue was processed and embedded in paraffin, Biowax blue (BioGnost, Zagreb, Croatia). Then, 5 µm thick sections were cut using a rotary microtome (Esselite Leitz, Stuttgart, Germany). Sections were deparaffinized and stained with hematoxylin and eosin for 2 min. Slides were mounted with coverslips and observed by light microscopy (Olympus BX51, Shinjuku, Tokyo, Japan).

### 2.10. Immunohistochemical Detection of Collagen Type I

Expression of osteogenic matrix marker collagen type I was chosen for immunohistochemical analysis to assess the osteogenic differentiation capacity of hMSCs cultured on the scaffolds. Sections were prepared and treated as described earlier [31]. Collagen type I detection process was carried out in the same manner as in our previous work [22], where a biological response was tested on magnesium substituted CaP samples.

Hereby, after 21 days of osteogenic differentiation, the ion substituted composite scaffolds were removed from the culture medium and prepared sections were incubated with primary antibody (anti-collagen I, Abcam, Cambridge, UK) and the signal was detected with EnVision Detection Systems Peroxidase/DAB, Rabbit/Mouse (Dako, Glostrup, Denmark), according to the manufacturer instructions. Scaffolds without cells but in the same culture conditions were used as blanks. Negative controls were processed in the same way with the omittance of the primary antibody. Human bone was used as a positive control. All slides were visualized using an Olympus BX51 microscope and images captured by a digital camera.

### 2.11. Isolation of Total RNA and RT-qPCR Analysis

Total RNA was collected from 21 days old tissue cultures using TRIzol reagent (Invitrogen Life Technologies, Sigma-Aldrich, Steinheim, Germany). Reverse transcription-quantitative real-time polymerase chain reaction (RT-qPCR) analysis was performed to quantify the expression of alkaline phosphatase (ALP) and dentin matrix protein 1 (DMP1). Analysis was performed according to manufacturer’s instructions and, in the same manner as in our previous work on magnesium substituted CaP scaffolds [22], to assess the osteogenic differentiation capacity of hMSCs cultured on composite scaffolds by detecting markers of characteristic osteogenic differentiation stages.

Power SYBR green PCR master mix (Applied Biosystems, Thermo Fisher Scientific, Waltham, MA, USA) was used to analyze the expression of commercially available primers (Sigma-Aldrich, Steinheim, Germany) for ALP and DMP1. RT-qPCR was performed on the 7500 Fast Real-Time PCR System (Applied Biosystems, Thermo Fisher Scientific, Waltham, MA, USA). Expression levels were normalized to *β*-actin. Relative expression of target genes was calculated using the ΔΔCt method.

### 2.12. Statistical Analysis

A one-way ANOVA test was employed to evaluate the significant differences between the two groups. The *p* < 0.05 and *p* < 0.01 values were considered statistically significant.

## 3. Results and Discussion

### 3.1. XRD Analysis and Whole Powder Pattern Decomposition Refinement

**Figure 1:**
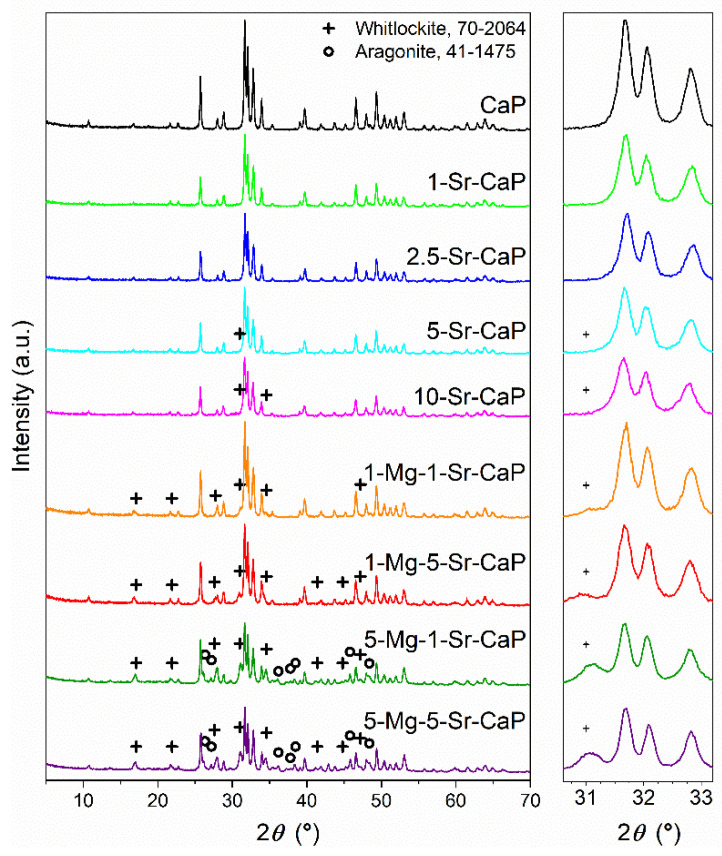
XRD patterns of prepared scaffolds. The 2*θ* positions of whitlockite and aragonite are marked with + and ○, respectively. The HAp (09-432) positions were not marked for clarity.

XRD patterns of Sr-substituted and Sr- and Mg co-substituted scaffolds are shown in Figure 1. Rietveld refinement analysis was performed on XRD patterns of all prepared samples to determine weight percent proportions of detected phases, unit–cell dimensions and crystallite size. Results of quantitative analysis obtained through whole powder pattern decomposition refinement ended with a resultant weighted profile factor, *R*_wp_, in the range between 6 and 9% (Table 2) indicating a good agreement between the calculated and the experimental data.

The XRD data for the samples CaP, 1-Sr-CaP and 2.5-Sr-CaP gave a good match to the line pattern for crystalline HAp (ICDD 09-432). In samples 5-Sr-CaP and 10-Sr-CaP in addition to HAp, a small amount (1–2%) of whitlockite (ICDD 70-2064) was identified as well (Table 2). The presence of the whitlockite phase is typical for Mg-substituted calcium phosphates and was not expected for Sr-substituted systems. It should be noted that biogenic calcium carbonate precursor (cuttlefish bone) contains Mg as a trace element, as confirmed by inductively coupled plasma mass spectrometry (ICP-MS) in the previous study [33]. Thus, it could be the reason for the presence of the whitlockite phase. Since WH is not identified in other scaffolds (CaP, 1-Sr-CaP and 2.5-Sr-CaP) we can only speculate that it eventually precipitates in a very small amount, that cannot be detected by XRD.

In all Sr-and Mg-co-substituted scaffolds the presence of HAp and WH phase was identified and for scaffolds 5-Mg-1-Sr-CaP and 5-Mg-5-Sr-CaP, in addition to HAp and WH patterns, low-intensity peaks with a good match to the diffraction lines of aragonite (ICDD 41-1475) were observed, as well.

As seen from Table 2, for co-substituted systems, with the increase in Mg^2+^ ion concentration in the starting reaction mixture a decrease in HAp content and an increase in the content of whitlockite and nontransformed aragonite were observed. The same trend we observed previously [20] for Mg-substituted systems.

In comparison to the sample 1-Mg-CaP, synthesized in our previous work [22], with the WH:HAp wt. ratio of 0.105, the samples 1-Mg-1-Sr-CaP and 1-Mg-5-Sr-CaP, with the WH:HAp wt. ratios of 0.106 and 0.137, respectively, show a slight increase in the WH phase with the increase in Sr ion concentration.

For the sample 5-Mg-CaP, the WH:HAp wt. ratio was 0.416 [22], and this value is fairly comparable to the results obtained for the samples 5-Mg-1-Sr-CaP and 5-Mg-5-Sr-CaP (0.435 and 0.400, respectively).

Unit cell parameters of HAp and WH phase obtained by the whole powder pattern decomposition refinements of the XRD data are given in Table 3.

As seen from Table 3a, the unit cell parameter *a* of HAp in the prepared Sr-substituted samples show no systematic change with increased levels of strontium while the unit cell parameter *c* and unit cell volume show a very slight (linear) increase. Compared to the non-substituted HAp (sample designated as CaP) in the sample 10-Sr-CaP, an increase in the parameter *c* of only 0.0074 Å was obtained. Such a subtle change at a low substitution percentage may be due to stabilization of hydroxyapatite structure that is simultaneously substituted with other ionic groups, such as CO_3_^2−^ and HPO_4_^2−^, which presence is confirmed by FTIR analysis.

To analyze the influence of strontium on the unit cell parameters of HAp and WH phase in the co-substituted samples 1-Mg-1-Sr-CaP and 1-Mg-5-Sr-CaP, unit cell parameters of the HAp and WH phase of the sample 1-Mg-CaP, corresponding to 1-Mg-0-Sr-CaP, reported in our previous paper [22], were used as references. In the same way for the co-substituted samples 5-Mg-1-Sr-CaP and 5-Mg-5-Sr-CaP, unit cell parameters of the HAp and WH phase of the sample 5-Mg-CaP, corresponding to 5-Mg-0-Sr, were used as references. It was observed that the lattice parameters of the HAp phase in co-substituted samples show very small and not systematic change with the increased level of strontium in the samples. However, the unit cell parameters and the unit cell volumes of the WH phase in co-substituted samples increased linearly with the increasing quantity of Sr^2+^. The latter results may indicate that Sr^2+^ ions, with greater ionic radius (1.12 Å) in comparison to Ca^2+^ (0.99 Å) have replaced Ca^2+^ sites in the WH phase. The observation is more pronounced for the samples with 1 mol.% of Mg^2+^. The increase in whitlockite unit cell parameters with magnesium and strontium co-substitution shows that strontium is incorporated into the whitlockite structure, has a significant effect on the increase in unit cell volume and it neutralizes the parameter reduction effect of magnesium during monosubstitution. An increase in the unit cell parameters of the magnesium-rich calcium phosphate phase during co-substitution with magnesium and strontium using precipitation in an aqueous medium was observed by Aina et al. [20] as well.

An energy-dispersive X-ray (EDX) analysis confirmed the presence of magnesium and strontium within the elemental composition of ion (co-)substituted scaffolds. The characteristic EDX spectra are presented in Appendix A.

### 3.2. FTIR Analysis

**Figure 2:**
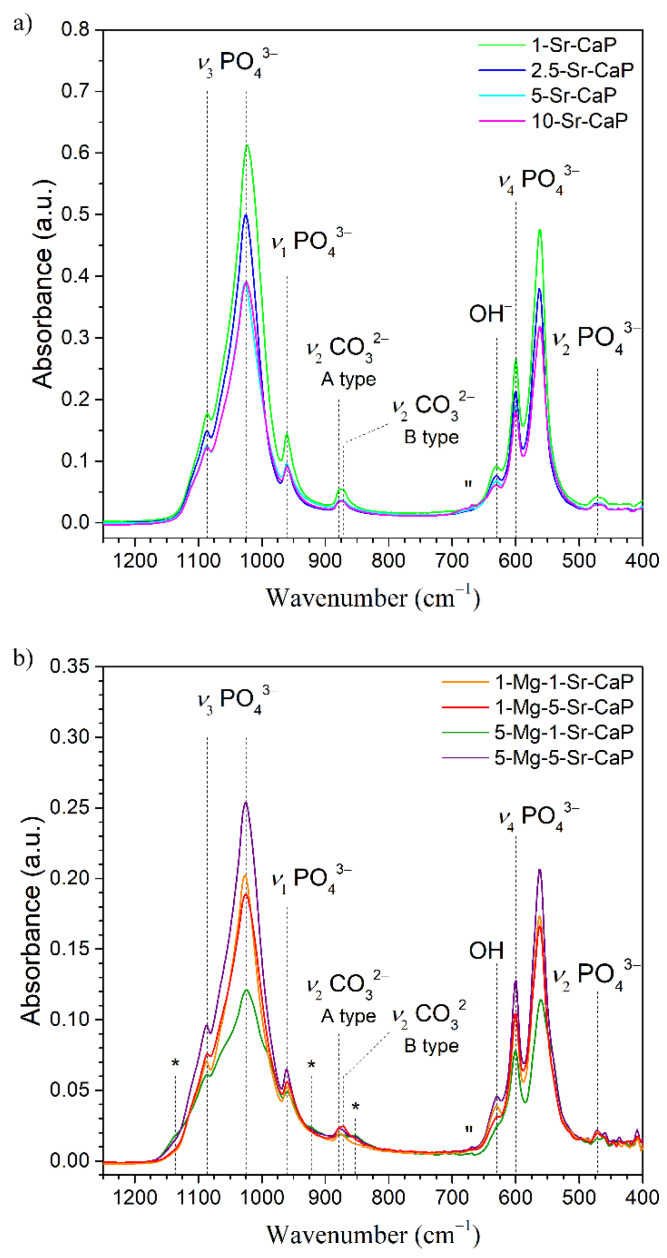
FTIR spectra of Sr-substituted (**a**) and Mg- and Sr-co-substituted (**b**) samples over the spectral range from 1250 to 400 cm^−1^. Appearance of low-intensity vibrational bands characteristic for whitlockite are marked with * and ″.

The Fourier transform infrared spectra of prepared samples are presented in Figure 2.

FTIR spectra of prepared samples show characteristic bands of HAp at 1086 and 1022 cm^−1^ (assigned to antisymmetric vibrations (ν_3_) of P-O bonds), 598 and 563 cm^−1^ (attributed to bending vibrations (ν_4_) of O-P-O groups), 963 cm^−1^ (due to symmetric vibration (ν_1_) of P-O bonds) and 473 cm^−1^ (assigned to ν_2_ bending vibration of PO_4_ units). OH^−^ vibration band is observed at 630 cm^−1^. The bands at 872 and 879 cm^−1^ are assigned to ν_2_ vibration mode of CO_3_^2−^ group characteristic for B-type (substitution for phosphate) and A-type (substitution for hydroxide) carbonate substitution, respectively [34,35]. The increase in substituted ion(s) content resulted in reduced intensities of all mentioned bands that can be related to the decrease in the relative amount of HAp phase in the samples. The appearance of low-intensity bands at 1136, 922 and 853 cm^−1^ (marked by * in Figure 2b) are in agreement with whitlockite formation [22], as confirmed by XRD. In the 10-Sr-CaP and Mg-Sr-CaP samples, the appearance of new bands at 670 cm^−1^ (marked by ″ in Figure 2b) were observed, in accordance with the observation reported by Aina et al. [20].

### 3.3. SEM Analysis

**Figure 3:**
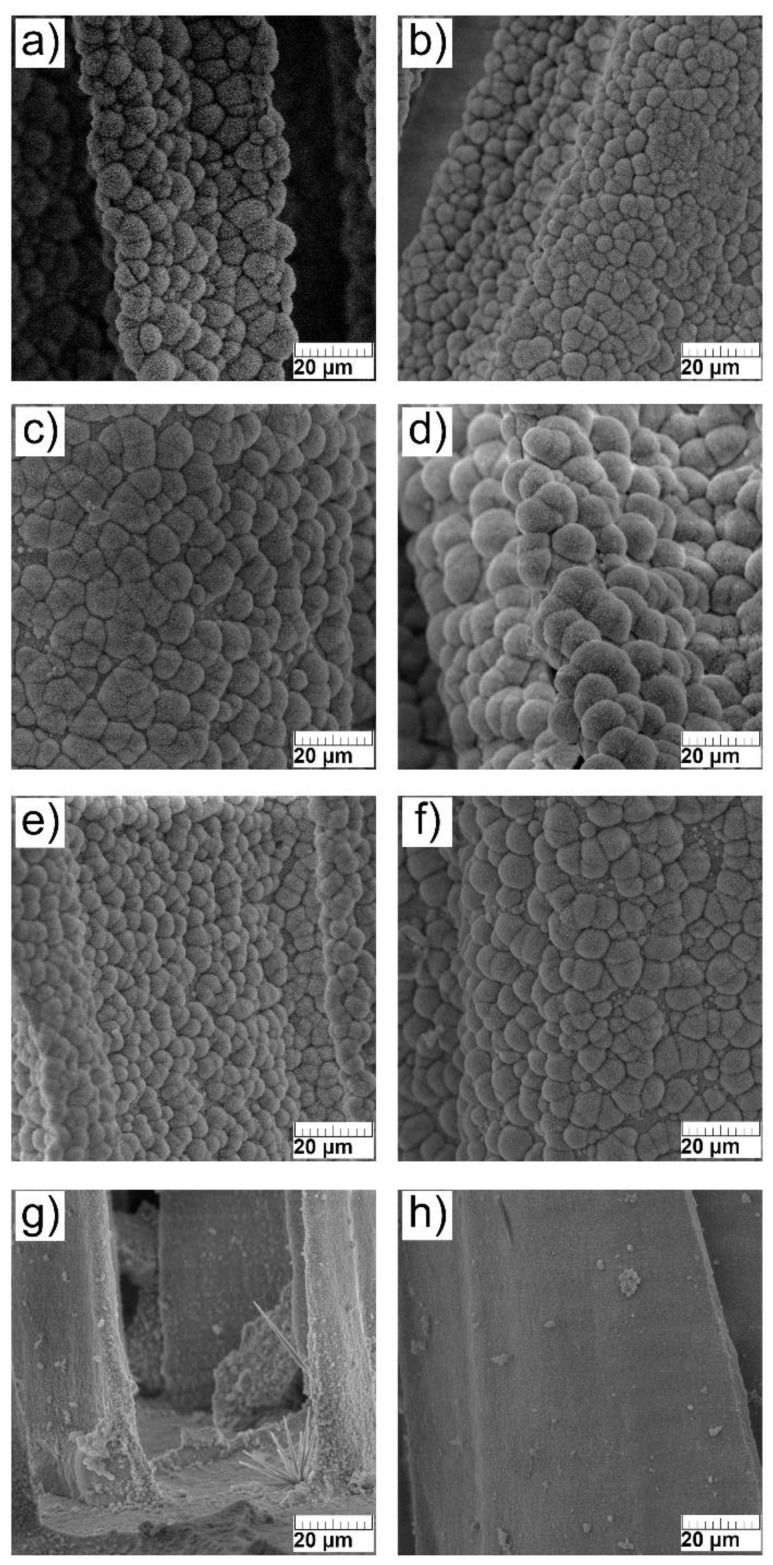
SEM micrographs of the surface of scaffolds: (**a**) 1-Sr-CaP, (**b**) 2.5-Sr-CaP, (**c**) 5-Sr-CaP, (**d**) 10-Sr-CaP, (**e**) 1-Mg-1-Sr-CaP, (**f**) 1-Mg-5-Sr-CaP, (**g**) 5-Mg-1-Sr-CaP, (**h**) 5-Mg-5-Sr-CaP. (scale bar: 20 µm).

The microstructure of the surface of prepared scaffolds was investigated by SEM analysis. In Figure 3a–f, irregularly shaped microspheres with cauliflower morphology, typical for HAp are observed.

As seen, the surface morphology of investigated scaffolds and the size of characteristic microspheres were not significantly affected by the addition of Sr^2+^ ions. The surface morphology of Mg- and Sr-co-substituted scaffolds 1-Mg-1-Sr-CaP and 1-Mg-5-Sr-CaP is comparable to that of Sr-substituted scaffolds. However, in the co-substituted scaffolds with the higher (5 mol.%) Mg^2+^ content, 5-Mg-1-Sr-CaP and 5-Mg-5-Sr-CaP, the surface morphology significantly changed, as clearly visible comparing Figure 3g,h to Figure 3a–f. The observation of a smoother surface of the latter samples is in accordance with our previous study [22] that showed that with the increase in Mg^2+^ content microspheres become smaller. Additionally, the formation of magnesium-rich crystals on readily exchangeable scaffold surfaces is in agreement with literature findings. Under biological conditions, magnesium tends to form a hydrated layer around hydroxyapatite crystals and thus influences surface behavior [18,36]. Samples co-substituted with 5 mol.% of Mg^2+^ contain around 11 mas.% of aragonite and 25–27 mas.% of whitlockite. Unfortunately, from SEM images it is not possible to distinguish between all detected phases (HAp, WH, aragonite).

It is worth noting that in the previous study of our research group [37], hydroxyapatite spheres with diameters from 3 to 8 µm were observed on the surface of lamellae, similar to those visible in the Figure 3a–f. At higher magnifications, dandelion-like structures of spheres were seen, with various radially oriented nanoplates and nanorods, with an average diameter of about 200–300 nm and an average length of about 8–10 µm [36]. These sizes are much larger than the crystallite size determined in this work by Rietveld refinements (from 50 to 70 nm for HAp, and from 17 to 25 nm for WH phase, Table 3) indicating that particles are made of several nanosized crystals.

### 3.4. Cytotoxicity Evaluation of Extracts

**Figure 4:**
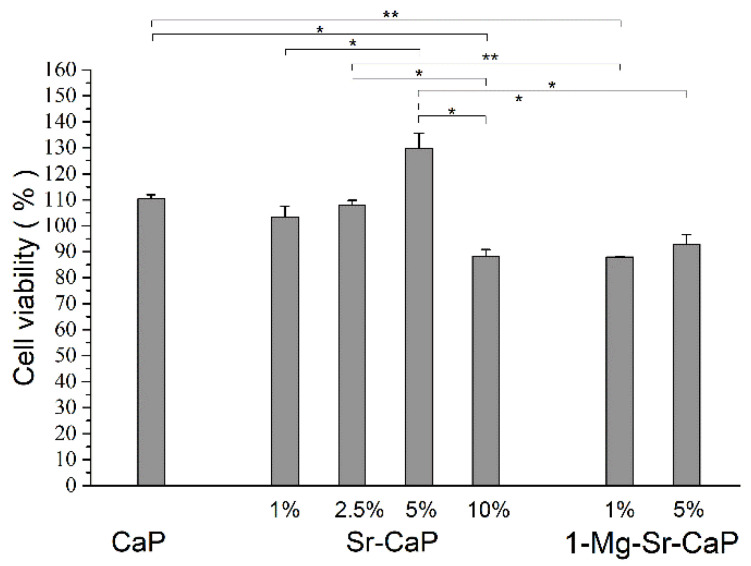
MTT assay of Hek293 human embryonic kidney cell line cultured in extracts of CaP, Sr-CaP and Mg-Sr-CaP samples for 72 h. Extracts were obtained after soaking the samples into the culture medium for 7 days. Data are expressed as a percentage over the control cells, considering the control group as 100% (untreated cells). The significant difference between two groups: * *p* < 0.05; ** *p* < 0.01.

The results of the MTT test are presented in Figure 4 as a percentage of viable Hek293 human embryonic kidney cells treated with sample extracts for 72 h. As shown before, an uncomplete hydrothermal conversion of aragonite was obtained in the Mg- and Sr-co-substituted scaffolds 5-Mg-1-Sr-CaP and 5-Mg-5-Sr-Ca and these samples were omitted in the cytotoxicity evaluation.

Estimated cells viability (between 85% and 130% of control) indicates that all studied scaffolds do not release toxic substances and do not show a negative effect on cell activity.

### 3.5. PCL-Coated Scaffolds

In previous papers of our research group [28,29] the influence of PCL impregnation on the mechanical and biological properties of hydroxyapatite scaffold derived from cuttlefish bone was studied.

Our previous study on Mg-substituted CaP porous scaffolds showed a positive impact of the whitlockite phase on the mechanical properties of scaffolds. Immunohistochemical staining showed that 1-Mg-CaP scaffolds with the HAp:WH wt. ratio of 90:10 exhibited higher expression of collagen type I and osteocalcin than pure HAp scaffold. As shown before, in the Mg- and Sr-co-substituted scaffolds 1-Mg-1-Sr-CaP and 1-Mg-5-Sr-CaP the HAp:WH wt. ratio was also cca. 90:10. Therefore the latter scaffolds were chosen for the preparation of composite materials with PCL. For comparison PCL-coated, nonsubstituted (CaP/PCL) and 1-Mg-CaP scaffolds were prepared as well.

The XRD diffraction patterns of composites (not shown), in addition to the diffraction peaks belonging to HAp and WH showed two additional strong diffraction peaks at Bragg angles 2*θ* = 21.3 and 23.8 attributed to the (110) and (200) crystallographic planes of semi-crystalline PCL [38]. FTIR spectra of prepared composites appeared as a superposition of the spectra of calcium phosphate phases and PCL. No other bands or band shifts were observed in the spectra indicating that no chemical reactions occurred between CaP and PCL. Results of thermogravimetric analysis of prepared composite scaffolds samples indicated that composites contain cca. 34 wt.% of PCL.

**Figure 5:**
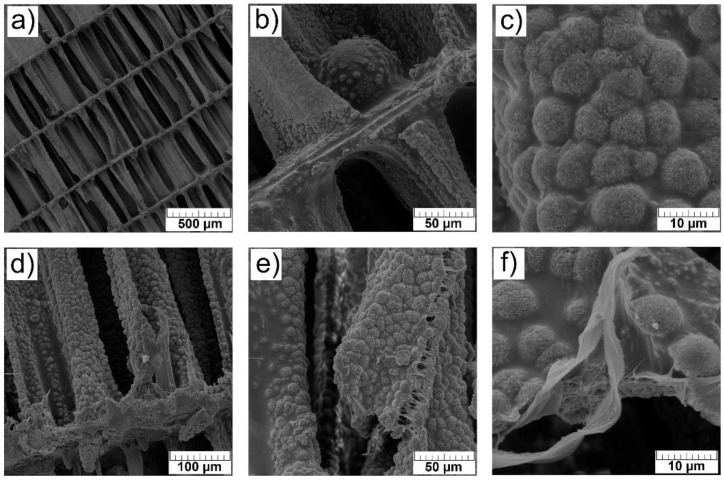
SEM micrographs of PCL coated 1-Mg-1-Sr-CaP scaffold. After polymer impregnation, the interconnectivity of the channels in the scaffold is maintained (**a**,**b**,**d**). The PCL layer on the irregularly shaped microspheres that resemble the cauliflower morphology is evident (**d**,**e**). The thin surface layer of PCL spread over the scaffold surface is visible on higher magnification (**c**,**f**).

Representative scanning electron micrographs of the composite scaffolds are shown in Figure 5.

As shown in Figure 5, the porous structure of cuttlefish bone, consisting of lamellae separated by numerous pillars, was maintained after the hydrothermal treatment and no blocking of pores after polymer impregnation was observed. It is well known that the interconnected porous structure is beneficial for bone growth and vascularization.

PCL layer evenly covers the surface of the irregularly shaped microspheres that resemble the cauliflower morphology. Some cracks (Figure 5e) and debonding between the PCL coating and inorganic CaP structures (Figure 5e) are seen, generated most probably during the preparation of samples (cutting) for SEM examination.

#### 3.5.1. Mechanical Properties

**Figure 6:**
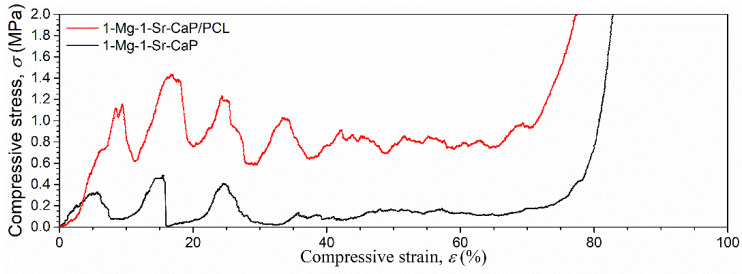
Typical compressive stress–strain curves for uncoated and PCL-coated scaffold.

To investigate the influence of PCL impregnation on the mechanical properties of porous CaP scaffolds compression tests were performed on CaP/PCL, 1-Mg-CaP/PCL, 1-Mg-1-Sr-CaP/PCL and 1-Mg-5-Sr-CaP/PCL composite specimens. For comparison, the corresponding uncoated CaP, 1-Mg-CaP, 1-Mg-1-Sr-CaP and 1-Mg-5-Sr-CaP scaffolds were tested as well. Typical compressive stress–strain curves for coated and uncoated specimens are shown in Figure 6.

**Figure 7:**
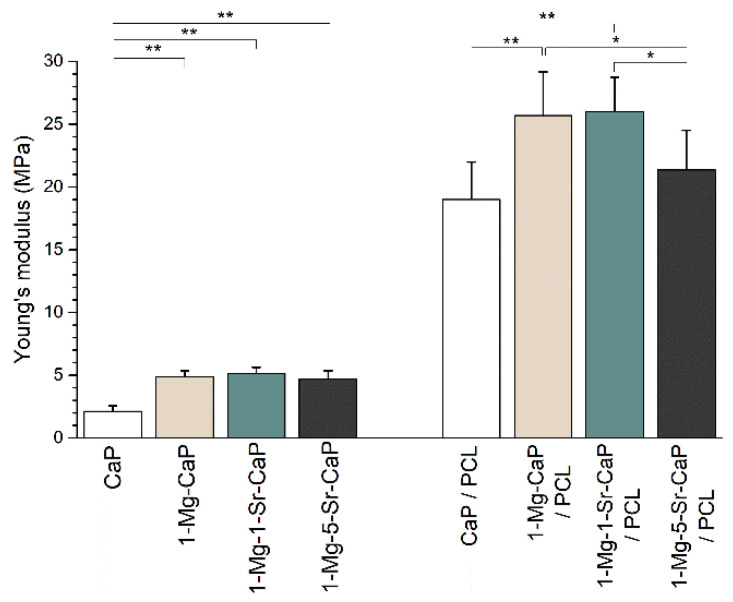
Young’s modulus of unsubstituted (CaP), ion substituted uncoated scaffolds (1-Mg-CaP, 1-Mg-1-Sr-CaP and 1-Mg-5-Sr-CaP) and corresponding coated scaffolds (1-Mg-CaP/PCL, 1-Mg-1-Sr-CaP/PCL and 1-Mg-5-Sr-CaP/PCL). At least nine replicas of each scaffold were tested and deviations are presented with error bars. The significant difference between two groups: * *p* < 0.05; ** *p* < 0.01.

The three different regions typical for porous structures [39] are seen, with the particular characteristic sawtooth behavior of cuttlefish-derived scaffolds in the pores’ collapse zone [28]. The initial increase in stress at low strain (linear-elastic region) is followed by a multi-peak profile due to the layer-by-layer collapse of the microstructure under compression. Further increase in load results in a densification region accompanied by a steep increase in stress where the uncoated specimens crumbled into powder. The compressive strength and elastic modulus of the porous scaffolds were quantified from the maximum stress and the initial slope of the stress–strain curve, respectively. The value of Young’s modulus of ion substituted uncoated scaffolds more than doubled compared to uncoated unsubstituted (CaP) scaffold (Figure 7).

For scaffolds CaP, 1-Mg-CaP, 1-Mg-1-Sr-CaP and 1-Mg-5-Sr-CaP values for Young’s moduli were 2.1 ± 0.5, 4.9 ± 0.5, 5.2 ± 0.4 and 4.7 ± 0.7 MPa, respectively. The increase could be ascribed to the presence of the whitlockite phase in ion substituted systems, in accordance with the results of Jang et al. [40] who found enhanced mechanical properties of WH bioceramic scaffolds in comparison with HAp scaffolds. As shown in Figure 7 PCL coating improved Young’s modulus of all investigated scaffolds, which increased more than five times compared to uncoated scaffolds, being 19.0 ± 3.0, 25.7 *±* 3.5, 26.0 ± 2.7 and 21.4 *±* 3.2 MPa for CaP/PCL, 1-Mg-CaP/PCL, 1-Mg-1-Sr-CaP/PCL and 1-Mg-5-Sr-CaP/PCL composite scaffold, respectively. The compressive strengths of uncoated scaffolds ranged between 0.20 and 0.35 MPa while coated scaffolds showed values higher than 1.1 MPa.

In the previous study reported by our research group [28,29], a higher concentration of PCL solution (20% *w*/*v*) was used to prepare a PCL-coated hydroxyapatite scaffold, compared to the concentration applied in this work (10% *w*/*v*). However, in spite of the smaller amount of polymer phase in the composite scaffold CaP/PCL prepared in this work (32.7 ± 1.8 wt.% vs. 48.7 ± 0.1 wt.% in [28]) mechanical properties were found slightly enhanced compared to previous results, where the elastic modulus of 15.5 ± 1.2 MPa and the compressive strength of 0.88 ± 0.11 MPa were determined [28].

The lower PCL concentration and solution viscosity are beneficial for the filling of the crack-like defects on the scaffold’s surface during vacuum infiltration, inhibiting crack propagation. Increasing the thickness of polymer coating, by increasing the concentration of polymer solution or by repeating the infiltration several times, could further enhance the mechanical properties of the scaffolds. However, it is unfavorable for the scaffold porosity and bioactivity since the coating will reduce or even impede the direct contact of the scaffold’s surface with the biological environment.

#### 3.5.2. Histological Analysis

**Figure 8:**
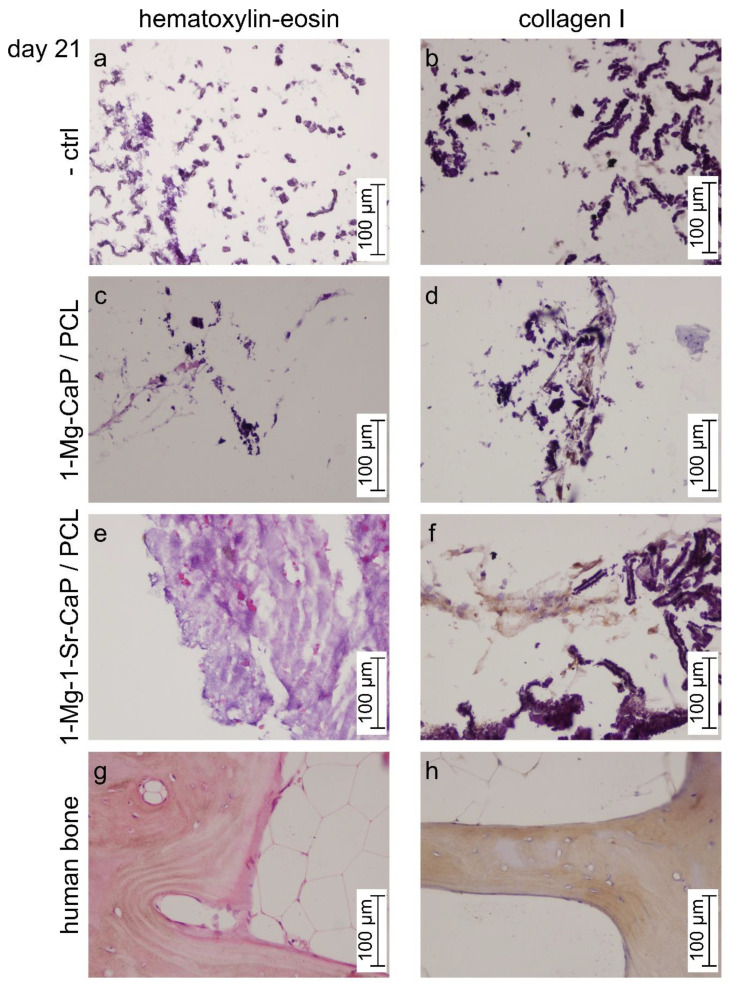
Histological staining (hematoxylin/eosin) and immunohistochemical examination of collagen type I on 1-Mg-CaP/PCL and 1-Mg-1-Sr-CaP/PCL scaffolds after 21 days of osteogenic induction. As negative control (-ctrl) sections were processed for each sample in the absence of the suitable antibody (**a**,**b**). Human bone was used as a sample for the positive control (**g**,**h**). In figure (**c**,**e**,**g**) nuclei are stained blue-purple, whereas the cytoplasm and extracellular matrix have varying degrees of pink staining. Brown color indicates positive staining of collagen (**b**,**d**,**f**). Representative images are shown at 20× magnification and the scale bars represent 100 μm.

To examine the osteogenic potential of prepared PCL-coated scaffolds the cell culture studies were performed in vitro with human mesenchymal stem cells (hMSCs) in an osteogenic medium for 21 days. To evaluate the effect of Mg^2+^ and Sr^2+^ ions co-substitution on the differentiation of hMSCs the composite scaffolds 1-Mg-CaP/PCL and 1-Mg-1-Sr-CaP/PCL were compared. Histological evaluation was performed with Hematoxylin and Eosin staining (H&E) and expression of collagen I was assessed by immunohistochemistry (Figure 8). Human bone was used as the positive control. In Figure 8g nuclei are stained blue-purple, whereas the cytoplasm and extracellular matrix (ECM) have varying degrees of pink staining. In Figure 8h brown positive staining indicates the presence of collagen I. Negative control sections of each sample (-crtl, Figure 8a,b) represents staining interference, originating from scaffold material, and should be excluded during the comparison.

Optical image of the cross-section of 1-Mg-CaP/PCL scaffold stained with H&E showed slightly pink-colored reticulate structures (Figure 8c, left part) with several more strongly colored pink dots, indicating the ECM formation. Much more dots distributed over the entire cross-section of 1-Mg-1-Sr-CaP/PCL scaffold can be seen (Figure 8e) indicating beneficial effects of Sr^2+^ ions on the tissue formation.

Collagen I expression (Figure 8d,f) is in concordance with the histology results. Compared to the 1-Mg-CaP/PCL, the scaffold 1-Mg-1-Sr-CaP/PCL exhibited stronger staining of collagen I, in a larger area of examined cross-section, indicating the cell-secreted ECM of interconnected network structure.

#### 3.5.3. Quantitative Evaluation of Osteoinduction

RT-qPCR is used to quantify the expression of osteoblast-related genes such as alkaline phosphatase (ALP) and dentin matrix protein 1 (DMP1). ALP is generally considered an early-stage marker for osteoblast phenotype and an important indicator of differentiation and mineralization [41]. DMP1, classified as a late marker of osteoinduction, is also thought to play an important role in tissue biomineralization [42]. In vitro, DMP1 acts as a hydroxyapatite crystal nucleator with very high calcium ion-binding capability [43] and binds specifically to type I collagen [44].

**Figure 9:**
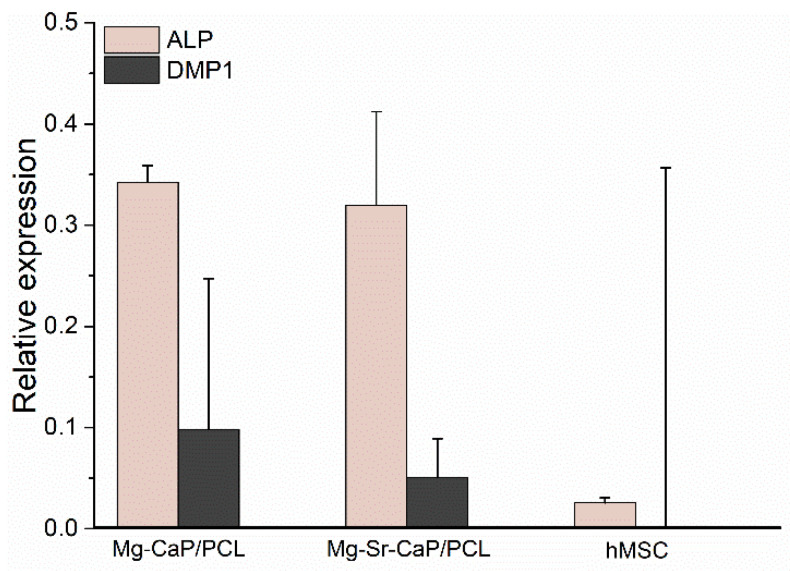
Relative expression of osteogenic markers on 1-Mg-CaP/PCL and 1-Mg-1-Sr-CaP/PCL scaffolds after 21 days of osteogenic induction. The relative gene expression was analyzed by the comparative cycle threshold method (ΔΔCt) and the values were normalized to *β*-actin expression. Quantified osteogenic markers were alkaline phosphatase (ALP) and dentin matrix protein 1 (DMP1). Undifferentiated hMSCs were used as a negative control, while human bone was used as a positive control (relative expression = 1).

As seen from Figure 9 both investigated composite scaffolds, 1-Mg-CaP and 1-Mg-1-Sr-CaP/PCL have expressed ALP and DMP1 and did not exhibit a significant difference in ALP and DMP1 expression after 21 days of culture.

In our previous paper [22] RT-qPCR analysis of uncoated CaP and Mg-CaP scaffolds showed higher expression of DMP1 compared to ALP expression, indicating later stages of osteoinduction. In this study, RT-qPCR analysis of PCL-coated scaffolds, showed a higher expression of ALP compared to DMP1 expression. Although the one-way ANOVA test evaluated statistically not significant differences between the ALP and DMP1 expression of the same scaffold, overall results indicate the retention of hMSC at an early stage of osteogenic differentiation. Further studies are needed to investigate the long-term behavior of prepared composite scaffolds in vitro. For a complete assessment of the biological performance in vivo studies should be carried out, as well.

## 4. Conclusions

In this study, a range of Sr-substituted and Mg- and Sr-co-substituted calcium phosphate (CaP) scaffolds, composed of carbonated hydroxyapatite and whitlockite, were prepared by hydrothermal conversion of cuttlefish bone. Furthermore, new composite scaffolds, combining biodegradable polymer-polycaprolactone (PCL) with ion-substituted scaffolds were fabricated. Mg^2+^ ions have a significant effect on the HAp/WH ratio and the surface appearance of scaffolds, while Sr^2+^ ions have a great influence on the WH unit cell parameters. In vitro cell culture confirmed that scaffolds are not cytotoxic. The compressive modulus of ion substituted scaffolds was more than doubled compared to nonsubstituted CaP scaffolds. The compressive modulus of PCL-coated scaffolds was found to be five times higher compared to uncoated scaffolds. The scaffold 1-Mg-1-Sr-CaP/PCL indicates stronger expression of collagen I, compared to the 1-Mg-CaP/PCL scaffold and could be a good candidate for bone tissue engineering applications.

## Figures and Tables

**Table 1:** Nominal composition and denomination of prepared samples.

Sample	Mg/(Ca + Mg + Sr) (mol.%)	Sr/(Ca + Mg + Sr) (mol.%)	(Ca + Mg + Sr)/P
CaP	0	0	10/6
1-Sr-CaP	0	1	10/6
2.5-Sr-CaP	0	2.5	10/6
5-Sr-CaP	0	5	10/6
10-Sr-CaP	0	10	10/6
1-Mg-1-Sr-CaP	1	1	10/6
1-Mg-5-Sr-CaP	1	5	10/6
5-Mg-1-Sr-CaP	5	1	10/6
5-Mg-5-Sr-CaP	5	5	10/6

**Table 2:** Results of quantitative analysis of samples obtained through whole powder pattern decomposition of XRD data. Resultant weighted factor, *R*_wp_, which indicates agreement between the calculated and the experimental data, of each refinement is shown in the last column.

Sample	wt.%			
	HAp	WH	Aragonite	*R*_wp_
CaP	100	0	0	5.98
1-Sr-CaP	100	0	0	5.948
2.5-Sr-CaP	100	0	0	5.976
5-Sr-CaP	99.07	0.93	0	6.441
10-Sr-CaP	97.92	2.08	0	6.556
1-Mg-1-Sr-CaP	90.39	9.61	0	6.788
1-Mg-5-Sr-CaP	87.96	12.04	0	6.977
5-Mg-1-Sr-CaP	61.78	26.90	11.32	7.789
5-Mg-5-Sr-CaP	63.19	25.29	11.52	8.295

**Table 3:** Unit cell parameters of the HAp and WH phase in Sr-substituted and Mg- and Sr-co-substituted systems obtained by the whole powder pattern decomposition refinements of the XRD data.

**(a)**				
**Sample**	**Hydroxyapatite**			
	**Unit Cell Paremeters (P6_3_/m)**			**Crystallite Size**
	***a*** **(Å)**	***c*** **(Å)**	**Volume** **(Å^3^)**	***L*** **_vol-IB_** **(nm)**
CaP	9.4330	6.8981	531.573	55.633
1-Sr-CaP	9.4351	6.9002	531.962	49.611
2.5-Sr-CaP	9.4305	6.9035	531.707	56.872
5-Sr-CaP	9.4316	6.9037	531.847	55.664
10-Sr-CaP	9.4323	6.9055	532.067	55.571
1-Mg-1-Sr-CaP	9.4317	6.9014	531.674	62.115
1-Mg-5-Sr-CaP	9.4369	6.9002	532.171	57.327
5-Mg-1-Sr-CaP	9.4328	6.8965	531.429	70.278
5-Mg-5-Sr-CaP	9.4345	6.8937	531.398	65.063
**(b)**				
**Sample**	**Whitlockite**			
	**Unit Cell Parameters (R3C)**			**Crystallite Size**
	***a*** **(Å)**	***c*** **(Å)**	**Volume** **(Å^3^)**	***L*** **_vol-IB_** **(nm)**
1-Mg-CaP	10.3795	37.2133	3472.044	24.680
5-Mg-CaP	10.3716	37.2386	3469.069	22.631
1-Mg-1-Sr-CaP	10.4021	37.2750	3492.903	19.489
1-Mg-5-Sr-CaP	10.4630	37.5080	3556.050	16.553
5-Mg-1-Sr-CaP	10.3720	37.2651	3471.265	18.486
5-Mg-5-Sr-CaP	10.3918	37.3312	3491.242	19.466

**Figure A1:**
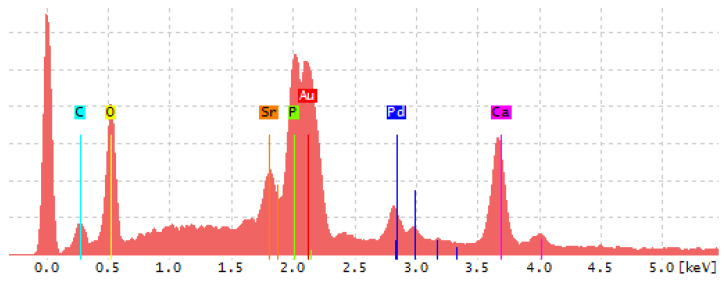
The characteristic energy dispersive X-ray analysis spectra showing peaks corresponding to the elements in the 5-Sr-CaP scaffold.

**Figure A2:**
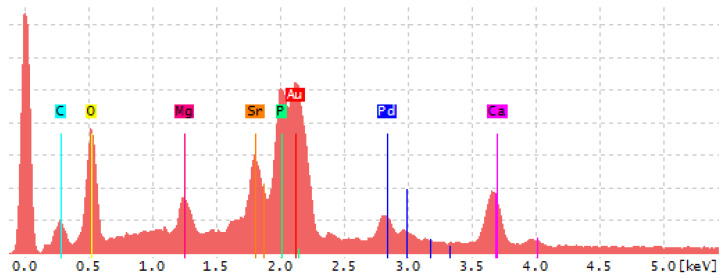
The characteristic energy dispersive X-ray analysis spectra showing peaks corresponding to the elements in the 5-Mg-5-Sr-CaP scaffold.

## Data Availability

Not applicable.

## Appendix A

The energy dispersive X-ray analysis has been used to confirm the atomic composition of prepared scaffolds. The appearance of gold and palladium in EDX spectra are the results of scaffold preparation with sputter coating for SEM-EDX analysis to obtain conductivity.

The EDX spectra of the 5-Sr-CaP scaffold (Figure A1) confirmed the presence of calcium, phosphorus, oxygen and strontium. In addition to the aforementioned atomic composition, the EDX spectra of the 5-Mg-5-Sr-CaP scaffold (Figure A2) confirmed the presence of magnesium. The characteristic EDX spectra of scaffolds with the highest substitution rate of magnesium and/or strontium are presented because spectral lines are the most distinguishable.

Synthesized samples are three-dimensional highly porous scaffolds consisting of two crystal phases (HAp and WH) with surface crystal agglomerates that change size and distribution pronouncedly with the addition of magnesium. Thus the EDX analysis, surface chemical microanalysis technique on selected point or limited area of interest, was not the appropriate method to confirm representable atomic weight composition of the prepared scaffolds.

## References

[1] Boanini E., Gazzano M., Bigi A. (2010). Ionic substitutions in calcium phosphates synthesized at low temperature. Acta Biomater..

[2] Shepherd J.H., Shepherd D.V., Best S.M. (2012). Substituted hydroxyapatites for bone repair. J. Mater. Sci. Mater. Med..

[3] Šupová M. (2015). Substituted hydroxyapatites for biomedical applications: A review. Ceram. Int..

[4] Tite T., Popa A.-C., Balescu L., Bogdan I., Pasuk I., Ferreira J., Stan G. (2018). Cationic Substitutions in Hydroxyapatite: Current Status of the Derived Biofunctional Effects and Their In Vitro Interrogation Methods. Materials.

[5] Landi E., Logroscino G., Proietti L., Tampieri A., Sandri M., Sprio S. (2008). Biomimetic Mg-substituted hydroxyapatite: From synthesis to in vivo behaviour. J. Mater. Sci. Mater. Med..

[6] Ren F., Leng Y., Xin R., Ge X. (2010). Synthesis, characterization and ab initio simulation of magnesium-substituted hydroxyapatite. Acta Biomater..

[7] Yuan X., Zhu B., Tong G., Su Y., Zhu X. (2013). Wet-chemical synthesis of Mg-doped hydroxyapatite nanoparticles by step reaction and ion exchange processes. J. Mater. Chem. B.

[8] Stipniece L., Salma-Ancane K., Borodajenko N., Sokolova M., Jakovlevs D., Berzina-Cimdina L. (2014). Characterization of Mg-substituted hydroxyapatite synthesized by wet chemical method. Ceram. Int..

[9] Li Z.Y., Lam W.M., Yang C., Xu B., Ni G.X., Abbah S.A., Cheung K.M.C., Luk K.D.K., Lu W.W. (2007). Chemical composition, crystal size and lattice structural changes after incorporation of strontium into biomimetic apatite. Biomaterials.

[10] Mardziah C.M., Sopyan I., Hamdi M., Ramesh S. (2008). Synthesis and characterization of strontium-doped hydroxyapatite powder via sol-gel method. Med. J. Malaysia.

[11] Ravi N.D., Balu R., Sampath Kumar T.S. (2012). Strontium-substituted calcium deficient hydroxyapatite nanoparticles: Synthesis, characterization, and antibacterial properties. J. Am. Ceram. Soc..

[12] Terra J., Dourado E.R., Eon J.G., Ellis D.E., Gonzalez G., Rossi A.M. (2009). The structure of strontium-doped hydroxyapatite: An experimental and theoretical study. Phys. Chem. Chem. Phys..

[13] Marie P.J. (2005). Strontium ranelate: A novel mode of action optimizing bone formation and resorption. Osteoporos. Int..

[14] Bonnelye E., Chabadel A., Saltel F., Jurdic P. (2008). Dual effect of strontium ranelate: Stimulation of osteoblast differentiation and inhibition of osteoclast formation and resorption in vitro. Bone.

[15] Bigi A., Boanini E., Capuccini C., Gazzano M. (2007). Strontium-substituted hydroxyapatite nanocrystals. Inorganica Chim. Acta.

[16] Zhu K., Yanagisawa K., Shimanouchi R., Onda A., Kajiyoshi K. (2006). Preferential occupancy of metal ions in the hydroxyapatite solid solutions synthesized by hydrothermal method. J. Eur. Ceram. Soc..

[17] Legeros R.Z., Kijkowska R., Bautista C., Legeros J.P. (1995). Synergistic effects of magnesium and carbonate on properties of biological and synthetic apatites. Connect. Tissue Res..

[18] Neuman W.F., Mulryan B.J. (1971). Synthetic hydroxyapatite crystals. Calcif. Tissue Res..

[19] Bigi A., Falini G., Foresti E., Ripamonti A., Gazzano M., Roveri N. (1993). Magnesium influence on hydroxyapatite crystallization. J. Inorg. Biochem..

[20] Aina V., Lusvardi G., Annaz B., Gibson I.R., Imrie F.E., Malavasi G., Menabue L., Cerrato G., Martra G. (2012). Magnesium- and strontium-co-substituted hydroxyapatite: The effects of doped-ions on the structure and chemico-physical properties. J. Mater. Sci. Mater. Med..

[21] Geng Z., Wang R., Li Z., Cui Z., Zhu S., Liang Y., Liu Y., Huijing B., Li X., Huo Q. (2016). Synthesis, characterization and biological evaluation of strontium/magnesium-co-substituted hydroxyapatite. J. Biomater. Appl..

[22] Bauer L., Antunović M., Rogina A., Ivanković M., Ivanković H. (2021). Bone-mimetic porous hydroxyapatite/whitlockite scaffolds: Preparation, characterization and interactions with human mesenchymal stem cells. J. Mater. Sci..

[23] (2014). TOPAS V5: General Profile and Structure Analysis Software for Powder Diffraction Data.

[24] Sudarsanan K., Young R.A. (1969). Significant precision in crystal structural details. Holly Springs hydroxyapatite. Acta Crystallogr. Sect. B Struct. Crystallogr. Cryst. Chem..

[25] Coelho A.A. (2000). Whole-profile structure solution from powder diffraction data using simulated annealing. J. Appl. Crystallogr..

[26] Gopal R., Calvo C., Ito J., Sabine W.K. (1974). Crystal Structure of Synthetic Mg-Whitlockite, Ca_18_Mg_2_H_2_(PO_4_)_14_. Can. J. Chem..

[27] Caspi E.N., Pokroy B., Lee P.L., Quintana J.P., Zolotoyabko E. (2005). On the structure of aragonite. Acta Crystallogr. Sect. B Struct. Sci..

[28] Milovac D., Gallego Ferrer G., Ivankovic M., Ivankovic H. (2014). PCL-coated hydroxyapatite scaffold derived from cuttlefish bone: Morphology, mechanical properties and bioactivity. Mater. Sci. Eng. C.

[29] Milovac D., Gamboa-Martínez T.C., Ivankovic M., Gallego Ferrer G., Ivankovic H. (2014). PCL-coated hydroxyapatite scaffold derived from cuttlefish bone: In vitro cell culture studies. Mater. Sci. Eng. C.

[30] Matic I., Antunovic M., Brkic S., Josipovic P., Caput Mihalic K., Karlak I., Ivkovic A., Marijanovic I. (2016). Expression of OCT-4 and SOX-2 in Bone Marrow-Derived Human Mesenchymal Stem Cells during Osteogenic Differentiation. Open Access Maced. J. Med. Sci..

[31] Rogina A., Antunović M., Pribolšan L., Caput Mihalić K., Vukasović A., Ivković A., Marijanović I., Gallego Ferrer G., Ivanković M., Ivanković H. (2017). Human Mesenchymal Stem Cells Differentiation Regulated by Hydroxyapatite Content within Chitosan-Based Scaffolds under Perfusion Conditions. Polymers.

[32] Panek M., Antunović M., Pribolšan L., Ivković A., Gotić M., Vukasović A., Caput Mihalić K., Pušić M., Jurkin T., Marijanović I. (2019). Bone Tissue Engineering in a Perfusion Bioreactor Using Dexamethasone-Loaded Peptide Hydrogel. Materials.

[33] Ressler A., Cvetnić M., Antunović M., Marijanović I., Ivanković M., Ivanković H. (2020). Strontium substituted biomimetic calcium phosphate system derived from cuttlefish bone. J. Biomed. Mater. Res. Part B Appl. Biomater..

[34] Landi E., Celotti G., Logroscino G., Tampieri A. (2003). Carbonated hydroxyapatite as bone substitute. J. Eur. Ceram. Soc..

[35] El Feki H., Rey C., Vignoles M. (1991). Carbonate ions in apatites: Infrared investigations in thev 4 CO_3_ domain. Calcif. Tissue Int..

[36] Mouriño V., Cattalini J.P., Boccaccini A.R. (2012). Metallic ions as therapeutic agents in tissue engineering scaffolds: An overview of their biological applications and strategies for new developments. J. R. Soc. Interface.

[37] Ivankovic H., Tkalcec E., Orlic S., Gallego Ferrer G., Schauperl Z. (2010). Hydroxyapatite formation from cuttlefish bones: Kinetics. J. Mater. Sci. Mater. Med..

[38] Chen E.-C., Wu T.-M. (2007). Isothermal crystallization kinetics and thermal behavior of poly(ε-caprolactone)/multi-walled carbon nanotube composites. Polym. Degrad. Stab..

[39] Gibson L.J., Ashby M.F. (1999). Cellular Solids: Structure and Properties.

[40] Jang H.L., Zheng G.B., Park J., Kim H.D., Baek H.-R., Lee H.K., Lee K., Han H.N., Lee C.-K., Hwang N.S. (2016). In Vitro and In Vivo Evaluation of Whitlockite Biocompatibility: Comparative Study with Hydroxyapatite and β -Tricalcium Phosphate. Adv. Healthc. Mater..

[41] Stucki U., Schmid J., Hämmerle C.F., Lang N.P. (2001). Temporal and local appearance of alkaline phosphatase activity in early stages of guided bone regeneration. Clin. Oral Implants Res..

[42] Rezai Rad M., Liu D., He H., Brooks H., Xiao M., Wise G.E., Yao S. (2015). The role of dentin matrix protein 1 (DMP1) in regulation of osteogenic differentiation of rat dental follicle stem cells (DFSCs). Arch. Oral Biol..

[43] He G., Dahl T., Veis A., George A. (2003). Nucleation of apatite crystals in vitro by self-assembled dentin matrix protein 1. Nat. Mater..

[44] He G., George A. (2004). Dentin Matrix Protein 1 Immobilized on Type I Collagen Fibrils Facilitates Apatite Deposition in Vitro. J. Biol. Chem..

